# Working Principle and Performance of a Scalable Gravimetric System for the Monitoring of Access to Public Places

**DOI:** 10.3390/s20247225

**Published:** 2020-12-17

**Authors:** Tommaso Addabbo, Ada Fort, Matteo Intravaia, Marco Mugnaini, Marco Tani, Valerio Vignoli, Stefano De Muro, Marco Tesei

**Affiliations:** 1 Department of Information Engineering and Mathematics, University of Siena, Via Roma 56, 53100 Siena, Italy; addabbo@diism.unisi.it (T.A.); ada@dii.unisi.it (A.F.); mugnaini@diism.unisi.it (M.M.); tani@diism.unisi.it (M.T.); vignoli@diism.unisi.it (V.V.); 2 Rete Ferroviaria Italiana S.p.A. Direzione Protezione Aziendale, Piazza della Croce Rossa 1, 00161 Roma, Italy; stfdemuro@gmail.com (S.D.M.); m.tesei@rfi.it (M.T.)

**Keywords:** access security, access monitoring, gravimetric system, gravimetric sensor, gravimetric platform

## Abstract

Here, we propose a novel application of a low-cost robust gravimetric system for public place access monitoring purposes. The proposed solution is intended to be exploited in a multi-sensor scenario, where heterogeneous information, coming from different sources (e.g., metal detectors and surveillance cameras), are collected in a central data fusion unit to obtain a more detailed and accurate evaluation of notable events. Specifically, the word “notable” refers essentially to two event categories: the first category is represented by irregular events, corresponding typically to multiple people passing together through a security gate; the second category includes some event subsets, whose notification can be interesting for assistance provision (in the case of people with disabilities), or for statistical analysis. The employed gravimetric sensor, compared to other devices existing in the literature, exhibits a simple scalable robust structure, made up of an array of rigid steel plates, each laid on four load cells. We developed a tailored hardware and software to individually acquire the load cell signals, and to post-process the data to formulate a classification of the notable events. The results are encouraging, showing a remarkable detectability of irregularities (95.3% of all the test cases) and a satisfactory identification of the other event types.

## 1. Introduction

Smart floors, footboards, and platforms are used in a large variety of applications. Traditionally, they are employed in the biomedical field, for postural or gait analysis [1,2,3,4,5] as an alternative to other techniques based on wearable sensors [6,7]. Furthermore, one of the main targets of the existing devices is related to the sensing, with high spatial resolution and high sensitivity, of the pressure field exerted by the feet of a patient standing, walking, or running. Technological solutions to this problem are usually offered by soft or flexible materials embedded within a large matrix of capacitive or piezoresistive pressure sensors, able to perform a dense spatial sampling of the sensitive device surface [2,3]. Despite the required high spatial resolution and sensitivity, in these applications, the robustness and cost usually are not critical issues: the smart floor is trodden in a controlled manner, at a moderate frequency, with the aim of analyzing the transit event in detail while performing complex, expensive and time-consuming examinations.

A different field of application for smart floors, footboards and platforms is security [8,9,10,11,12,13,14,15,16,17,18]. Herein, the floor is used to monitor the occupancy of surveilled or sensitive areas, possibly to identify the people present inside these areas and, finally, in the novel application field considered in this paper, for the control of access in walk-through gates.

Some of the solutions present in the literature exploit the same typology of devices as those developed for the biomedical field, accompanying them with specific signal processing strategies [8,9]. Actually, however, the requirements for this kind of application, in most cases, are different from those listed previously concerning the biomedical field. Truthfully, in the context of security or access control, two very critical issues are the robustness and cost of the device, whereas high spatial resolution and sensitivity can be considered less essential. This is especially true for the application proposed in this paper, i.e., automatic control of accesses in public places through walk-through gates, where a very high throughput is expected, and fast (theoretically on-line) analysis of transit events is needed. The technology proposed to meet the requirements of this specific application is provided by gravimetric systems: in fact, different from sensor matrix-based footboards, gravimetric platforms have a simpler but more robust structure, similar to the one of a common weighing scale, consisting of a rigid plate supported by a small number of force sensors (such as load cells), usually placed at the platform corners [17,18]. Obviously, the platform surface is not an active sensing area; nevertheless, the output of the force sensors besides providing information about the magnitude of the net vertical force applied to the plate, can be used to accurately assess its application point position [17,18] as a function of time. When only one person or object is present on the footboard, her/his/its position can be estimated quite simply and accurately, as shown in [18], where this approach is applied to the localization of a robot. Things get more complicated if more than one person is present on the gravimetric platform, or if one person is pulling a luggage on wheels, or, more generally speaking, if there are other application points of the vertical forces beyond the feet of a single person [17]. Indeed, the force information is integrated over the relatively large area of the plate and this limits the capability of spotting any type of irregularities.

Here, we analyze the working principle and the performance of a scalable smart gravimetric system, described in a previous paper by the same authors [17], which is specifically designed for the control of walk-through accesses to public places with the aim of detecting simultaneous passages of more than one person. The system is based on a gravimetric footboard composed of a small array of traditional gravimetric platforms [17]. The proposed system is conceived to operate together with other deployed systems, such as surveillance cameras, metal detectors and Radio Frequency Identification (RFID) readers. This data fusion approach, brought to the fore by the spreading paradigm of the Internet of Things (IoT), can lead to a more detailed and trustable characterization of events or phenomena of interest [19]. Therefore, the main purpose is to add a further security level, which coincides, in the case of places with an entrance pass required, with the detection of the contemporary presence on the footboard of more than one person trying to get in the area under observation using only one ticket. Conversely, the proposed gravimetric system can be used to detect not irregular but still relevant events, such as the passage of a person with a luggage on wheels, with a stroller, or a person in a wheelchair or on crutches. Along these lines, the appropriate assistance could be arranged more quickly and effectively. Furthermore, this kind of data can be useful for the statistical characterization of the monitored place frequentation.

This manuscript is organized as follows: Section 2 describes the proposed gravimetric system working principle from a general theoretical perspective, also providing the derivation of the net force application point position uncertainty; in Section 3 the effectiveness and the performance of the proposed solution are investigated by means of experiments and tests. To this end, a prototype of the gravimetric sensor array is used together with a tailored laboratory acquisition system; Section 4 contains comments and further details on the results presented in Section 3. Section 5 presents the overall final gravimetric system, comprising the gravimetric sensor and the embedded electronics. Finally, Section 6 draws the conclusions.

### State of the Art

The technological evolution of gravimetric sensors is pushing toward integrable, scalable, and pervasive but non-intrusive solutions. Regarding this, slim and flexible sensor matrices, that can be embedded into the floor with no encumbrance for human activities, represent one of the latest research fields. Different sensor types are employed: the more common and recent proposals, that can be found in the literature, involve capacitive sensors [16] and piezoresistive Polymer Thick Films (PTFs) [8,9,10,11,14]. A more advanced piezoresistive carbon nanotube polymer sensor matrix is presented in [20]. The authors introduce a smart sensing floor based on triboelectricity in [4]. This effect also is used in other interesting research lines, where smart floors are even depicted as possible energy harvesters: for example, triboelectric nanogenerators (TENGs) are inserted in the floor to exploit the triboelectric effect both for motion sensing and for collecting energy from low-frequency mechanical vibrations [21].

As already stated in the previous section, the novel application introduced in this article, that is the monitoring of the access to public places, poses some serious challenges in terms of durability and reliability, since the system should be able to sustain a high throughput (many passages per day) without undergoing structural faults. Conversely, sensor integrability and size are less restrictive constraints with respect to domestic, office or biomedical applications. Thus, we opted for a simpler but more robust architecture, like the one proposed in [18], that we already introduced in our previous work [17].

Concerning data processing, the more advanced techniques include Fisher discriminant analysis [8], Support Vector Machines [13], and Convolutional Neural Networks (CNN) [4,15].

## 2. The Gravimetric System Working Principle

### 2.1. Gravimetric Sensor: Structure

A simple and robust realization of a gravimetric platform consists of a rigid plate laid on force sensors, with a vertical sensitivity axis, as already mentioned in the Introduction. There are usually four sensors placed at the platform corners. This configuration allows them to obtain the weight and the trajectory of a body moving upon the platform; as a disadvantage, it permits neither the extraction of detailed pressure maps for gait or postural analysis, nor the straightforward recognition of multiple objects on the platform, since the information provided by the platform is related to the motion of a single center of mass. Nevertheless, the usage of an array of gravimetric platforms can enhance the distinction capabilities when different objects are located on different elements of the array.

**Figure 1:**
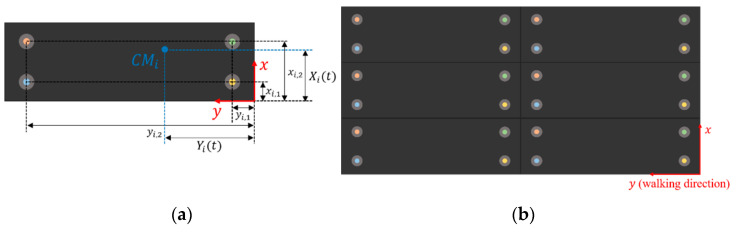
(**a**) Single gravimetric platform schematic; (**b**) Total gravimetric sensor (footboard) schematic.

Figure 1a shows a schematic of a single gravimetric platform with four force sensors (e.g., load cells) located near the four corners and individuated by $x_{i,1},\;x_{i,2},\;y_{i,1},\;y_{i,2}$ coordinates with respect to one corner. The i subscript is the platform index, used later to extend the calculations to the generic case of an array of N platforms. A gravimetric footboard can be obtained as an array of N identical platforms as in Figure 1b, which shows a possible disposition of gravimetric platforms.

The idea at the basis of the extension of the sensor into an array is to improve the capability of detecting the passage of more than one person at a time on the footboard. Therefore, the width of the single platform can be optimally selected so as to maximize the probability that only one person at time can comfortably pass on the single platform, whereas the length can be selected as to accommodate a single average step.

### 2.2. Gravimetric Sensor: Load and Trajectory Estimation

The magnitude $M_{i}\left(t\right)$ of the net vertical force applied on the i-th footboard at the time t can be obtained by the sum of the forces $m_{i,j}\left(t\right)$ acting on each force sensor and measured by the sensors themselves:

(1) $$M_{i}\left(t\right)=\overset{4}{\underset{j=1}{\sum }}m_{i,j}\left(t\right),$$

where j is the sensor index. Note that the resultant force $M_{i}\left(t\right)$ is the product of the inertial mass of the object and the vertical acceleration and, in static conditions, coincides with the weight of the object; therefore, in this paper we sometimes refer to this quantity with the term weight or load.

The extraction of object trajectories on a single gravimetric platform is plainly based on torque equilibrium calculations. The location coordinates $X_{i}\left(t\right)$, $Y_{i}\left(t\right)$ of the net force application point on the i-th footboard at the time t can be inferred applying torque equilibrium with respect to the corner chosen as a reference:

(2) $$X_{i}\left(t\right)=\frac{\sum_{j=1}^{4}x_{i,j}m_{i,j}\left(t\right)}{M_{i}\left(t\right)};\;Y_{i}\left(t\right)=\frac{\sum_{j=1}^{4}y_{i,j}m_{i,j}\left(t\right)}{M_{i}\left(t\right)}.$$

**Figure 2:**
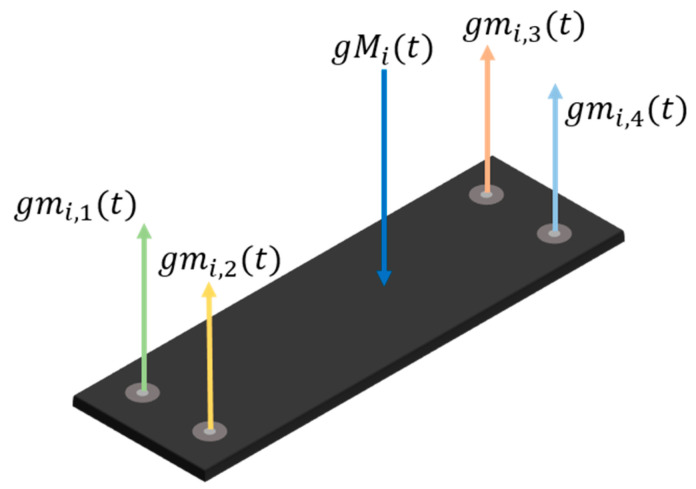
Constraint reactions of the load cells and total applied force.

Figure 2 gives an explanatory visualization of Equation (2), showing the constraint reactions opposing the total force acting on the footboard and measured by the force sensors.

Let now us consider a two-dimensional array of N gravimetric platforms. Then, the total force M(t) acting on the array at the time t is:

(3) $$M\left(t\right)=\overset{N}{\underset{i=1}{\sum }}M_{i}\left(t\right),$$

and the position of the total force application point on the platform at the time t, given by the X(t), Y(t) coordinates, can be written as follows:

(4) $$X\left(t\right)=\frac{\sum_{i=1}^{N}X_{i}\left(t\right)M_{i}\left(t\right)}{M\left(t\right)};\;Y\left(t\right)=\frac{\sum_{i=1}^{N}Y_{i}\left(t\right)M_{i}\left(t\right)}{M\left(t\right)}.$$

### 2.3. Performance of the Gravimetric System: Uncertainty

An important aspect to be considered is the accuracy with which a system such the one described in Section 2.1 is capable of determining forces and trajectories by applying Equations (1)–(4).

To assess this performance, we used the well-known propagation formula of uncertainty for indirect measurement under the assumption of statistically independent errors, which is based on the evaluation of the variance. Assuming the measurand ζ related through the function f to k quantities $\xi_{i}$, i.e., $\zeta =f\left(\xi_{1},\;\xi_{2},\;\ldots ,\;\xi_{k}\right)$, for small errors we can write:

(5) $$\sigma_{\zeta }^{2}=\overset{k}{\underset{i=1}{\sum }}{\left(\frac{\partial f}{\partial \xi_{i}}\right)}^{2}\sigma_{\xi_{i}}^{2},$$

where $\sigma_{\xi_{i}}^{2}$ are the standard deviations of the measurement error of $\xi_{i}$, whereas $\sigma_{\zeta }^{2}$ is the resulting standard deviation of the measurement error of ζ.

This formula, which concerns variances, is straightforwardly exploited to derive the uncertainties replacing all the standard deviations with their estimations (uncertainties) and is used for the measured weights and the trajectories.

The statistical independence assumption is supported by the physical separation of the acquisition of each sensor signal. Regarding the following, the force sensors are supposed as identical, characterized by measurement noise modeled by white ergodic stationary stochastic processes independent and identically distributed, with zero mean, therefore characterized by the same time-invariant uncertainty u(m). Likewise, the errors on the positioning of all sensors along the two axes are considered independent identically distributed random variables, with zero mean, characterized by the uncertainties u(x) and u(y), respectively.

The application of Equation (5) for uncertainty evaluation to the case of a single platform yields:

(6) $$u\left(M_{i}\right)=2u\left(m\right)$$

(7) $$u\left(X_{i}\left(t\right)\right)=\sqrt{\overset{4}{\underset{j=1}{\sum }}{\left(\frac{m_{i,j}\left(t\right)}{M_{i}\left(t\right)}u\left(x\right)\right)}^{2}+\overset{4}{\underset{j=1}{\sum }}{\left(\frac{x_{i,j}M_{i}\left(t\right)-\sum_{k=1}^{4}x_{i,k}m_{i,k}\left(t\right)}{{\left(M_{i}\left(t\right)\right)}^{2}}u\left(m\right)\right)}^{2}};\;u\left(Y_{i}\left(t\right)\right)=\sqrt{\overset{4}{\underset{j=1}{\sum }}{\left(\frac{m_{i,j}\left(t\right)}{M_{i}\left(t\right)}u\left(y\right)\right)}^{2}+\overset{4}{\underset{j=1}{\sum }}{\left(\frac{y_{i,j}M_{i}\left(t\right)-\sum_{k=1}^{4}y_{i,k}m_{i,k}\left(t\right)}{{\left(M_{i}\left(t\right)\right)}^{2}}u\left(m\right)\right)}^{2}},$$

where $u\left(X_{i}\left(t\right)\right)$ and $u\left(Y_{i}\left(t\right)\right)$ are the uncertainties on the trajectory coordinates on the i-th platform, whereas $u\left(M_{i}\right)$ indicates the uncertainty of the measured load on each platform.

It can be seen that the uncertainty of the trajectory estimation varies depending on the load application point position. Considering, as an example, the uncertainty evaluated for $X_{i}\left(t\right)$, it can be easily shown that:

(8) $$u\left(X_{i}\left(t\right)\right)<\sqrt{u{\left(x\right)}^{2}+{\left(\frac{2w}{M_{i}\left(t\right)}\right)}^{2}u{\left(m\right)}^{2}}$$

Note that $w=x_{i,2}-x_{i,1}$ is the distance between the sensors along the x direction. Therefore, the estimation of the x coordinate becomes critical when the measured load signal becomes small (very low load on the platform). These situations can be easily excluded setting a load threshold for platform operations, $M_{imin}$, that is chosen as the minimum significant load for the proposed application (e.g., 10 N corresponding to a standing mass equal to 10 kg). Below this threshold the signal from the force sensors are considered only noise and are not processed.

Therefore, the worst-case uncertainty can be evaluated:

(9) $$u\left(X_{i}\left(t\right)\right)<\sqrt{u{\left(x\right)}^{2}+4w^{2}{\left(\frac{u\left(m\right)}{M_{imin}}\right)}^{2}}=\sqrt{u{\left(x\right)}^{2}+4w^{2}u_{R_{MAX}}{\left(m\right)}^{2}}$$

Equation (9) clarifies that the coordinate estimation accuracy is limited by the maximum accepted relative load error, $u_{R_{MAX}}\left(m\right)$. This discussion can be easily extended to $u\left(Y_{i}\left(t\right)\right)$. More in general, given a certain platform, i.e., once the force sensors are chosen, as well as the platform geometry and the load sensor positioning tolerances, the minimum load threshold for trajectory calculation can be selected to guarantee a given level of accuracy.

The extension to the N footboard array case is straightforward:

(10) $$u\left(M_{i}\right)=2\sqrt{N}u\left(m\right)$$

(11) $$u\left(X\left(t\right)\right)=\sqrt{\overset{N}{\underset{i=1}{\sum }}{\left(\frac{M_{i}\left(t\right)}{M\left(t\right)}u\left(X_{i}\left(t\right)\right)\right)}^{2}+\overset{N}{\underset{i=1}{\sum }}{\left(\frac{X_{i}\left(t\right)M\left(t\right)-\sum_{k=1}^{N}X_{k}\left(t\right)M_{k}\left(t\right)}{{\left(M\left(t\right)\right)}^{2}}u(M_{i})\right)}^{2}};\;u\left(X\left(t\right)\right)=\sqrt{\overset{N}{\underset{i=1}{\sum }}{\left(\frac{M_{i}\left(t\right)}{M\left(t\right)}u\left(Y_{i}\left(t\right)\right)\right)}^{2}+\overset{N}{\underset{i=1}{\sum }}{\left(\frac{Y_{i}\left(t\right)M\left(t\right)-\sum_{k=1}^{N}Y_{k}\left(t\right)M_{k}\left(t\right)}{{\left(M\left(t\right)\right)}^{2}}u(M_{i})\right)}^{2}}.$$

**Figure 3:**
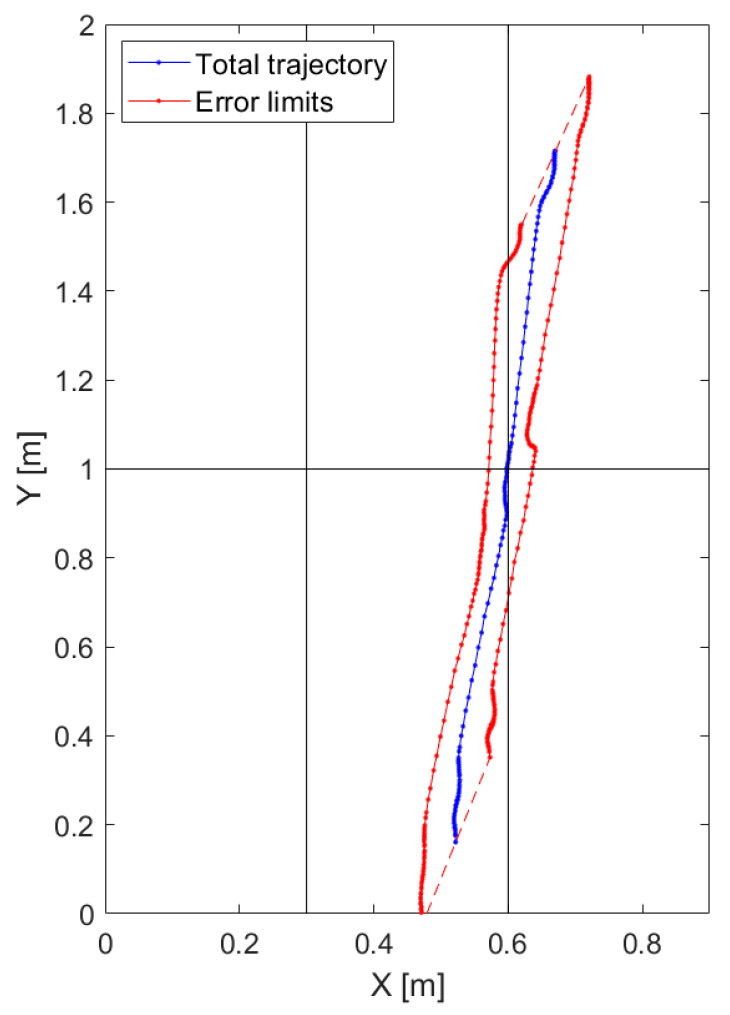
Simulated trajectory (blue) on a 2 × 0.9 m^2^ footboard with error band (red).

Considering plausible values for the sensor position errors (≈5 mm), and force sensors with a ∼2 N peak-to-peak noise value and a signal full range of 2000 N, a gravimetric footboard size in the range (2 ÷ 3 m^2^) and a minimum load of ten of kilograms, a satisfactory trajectory estimation, with an uncertainty in the order of some centimeters, is achievable. Figure 3 shows a simulated trajectory (in blue) on a gravimetric footboard of 2 × 0.9 m^2^, with the error bounds (in red) resulting from evaluating Equation (11).

### 2.4. Data Processing

The capability of the proposed system of recognizing different situations is obtained exploiting the equations reported in Section 2.1, allowing the estimation of loads and trajectories on the single platforms and loads and trajectory on the complete footboard.

The different situations (test cases) chosen in this paper for assessing the gravimetric system applicability are typical occurrences in an access monitoring scenario. Particularly, we considered the following event categories: single person without any particular object (“standard event” category), single person with luggage on wheels or with a stroller (grouped in a “wheel event” category), single person on crutches (“crutches event” category), two people side-by-side or two or more people queued (grouped in an “irregular event” category). The first three categories do not represent a security violation and, therefore, they are eventually grouped in a generic “regular event” category, as opposed to the irregular event category.

**Figure 4:**
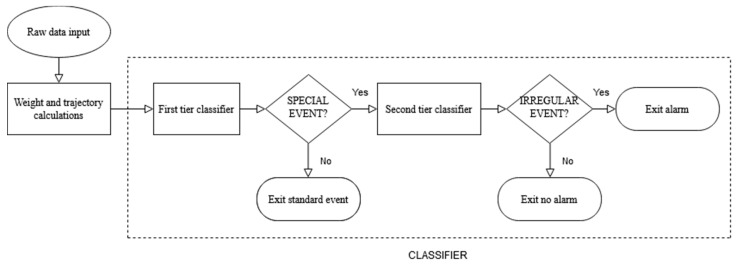
Data processing flow chart.

The data processing can be subdivided into two phases: the load and trajectory calculation and preprocessing, and the event recognition. During the first phase, Equations (1)–(4) are applied to the analog to digital (A/D) converted force sensor output signals ($m_{i,j}\left(k\right),\;i=1,\ldots ,\;N;\;j=1,\ldots ,\;4;\;k\in \mathbb{N}$, such that $M_{i}\left(k\right)>M_{imin}$) when the load on the single platform is above the predetermined threshold, $M_{imin}$, and low-pass filtered, to produce the set of discrete signals $\left\{M_{i}\left(k\right),\;X_{i}\left(k\right),\;Y_{i}\left(k\right),\;M\left(k\right),\;X\left(k\right),Y\left(k\right)\right\},\;i=1,\ldots ,N,\;k\in \mathbb{N}$ sampling the trajectory of the person who crossed the platform. Trajectory and load data are the input for the second part of the processing, namely the event recognition algorithm. The proposed recognition strategy is a multi-layer approach with a two-tier structure. The first level only separates standard events (i.e., the passage of a single person without any particular object) from all the other event categories, which are collectively named “special events” at this step. Concerning the case of a standard event, no warning is generated, and the algorithm exits. Conversely, in the case of a special event, the analysis continues with the second level event recognition algorithm. This second tier can generate either an alarm (in the case of irregularities, i.e., more than one person passing together on the platform), or only a notification (in the other cases), always providing a label indicating the hypothesized category for the occurred event. The first and second levels of the event recognition algorithm are described in detail in Section 2.4.1 and Section 2.4.2, respectively. Figure 4 shows a flow chart summarizing the whole algorithm working logic.

#### 2.4.1. First Tier Event Recognition Algorithm

The first level of the event recognition algorithm is based on the analysis of the two coordinates X(k) and Y(k), the transit direction, which are processed separately. The coordinate Y(k) is analyzed to detect the so-called ‘back steps’. Indeed, during a usual regular event of a walk-through, this coordinate is a non-decreasing function of time; only if the passing person decides to invert the walking direction on the footboard, the coordinate slope changes sign. Conversely, a second person stepping on the platform suddenly adds a load applied close to the footboard edge (Y(k) = 0 m), with the consequence of a displacement of the net force application point toward this edge, resulting in a change of the slope sign of Y(k). When a ‘back step’ event is found, a first net force average is evaluated using the *n* samples preceding the event (with *n* at least equal to ten), and a second average is calculated using the *n* samples following it. These two values are verified to be close within a certain threshold; if this is true, then the event is considered a real and regular back-step, i.e., the passage is regular but the single person on the footboard decides to invert the transit direction. Conversely, if the two average values difference exceeds the preset threshold, then the algorithm associates the back step to the arrival of a second person on the platform and sets a special event flag, passing the execution to the second-tier event recognition algorithm.

Regarding the X(k) coordinate, the algorithm seeks in every sample the maximum distance between the application points of the net force relative to the loaded platforms. As in the previous case, when this distance exceeds an appropriate threshold, the algorithm sets the special event flag, as it is likely that two people side-by-side are crossing the platform. This processing applied to the X coordinate is useful when the platform width is chosen as described in Section 2.1, i.e., granting a high probability that only one person at time can comfortably pass on the single platform.

#### 2.4.2. Second Tier Event Recognition Algorithm

The second-tier event recognition algorithm is based on a particular feature extraction. The algorithm is capable of extracting people, wheel (e.g., a stroller or luggage on wheels), and pressure spot (e.g., crutches or walking sticks) signals, considering force magnitude and distribution. Particularly, the process of separating people tracks from wheel tracks makes use only of the force magnitude information, while the force distribution information is added for isolating pressure spot tracks. The effect of this procedure will be clear in the next section, comparing Figures 6a, 9 and 10a,c. Fully, a number C=3 of trajectory types is considered. The output of this algorithm is a larger set of trajectories and loads of the above listed types. Therefore, the input of the algorithm is a set of N+1 trajectories and loads, $\left\{M_{i}\left(k\right),\;X_{i}\left(k\right),\;Y_{i}\left(k\right),\;M\left(k\right),\;X\left(k\right),Y\left(k\right)\right\},\;i=1,\ldots ,N,\;k\in \mathbb{N},$ whereas its output consists of (N+1)·C trajectories and loads, i.e., $\left\{M_{i,c},\left(k_{c}\right),\;X_{i,c}\left(k_{c}\right),\;Y_{i,c}\left(k_{c}\right),\;M_{c}\left(k_{c}\right),\;X_{c}\left(k_{c}\right),Y_{c}\left(k_{c}\right)\right\},\;c=people,\;wheel,\;spot,\;i=1,\ldots ,N,\;k_{c}\in \mathbb{N}$ such that $M_{cMax}>M_{i}\left(k_{c}\right)>M_{cMin}$. This means that the trajectories $X_{i,c}\left(k_{c}\right),\;Y_{i,c}\left(k_{c}\right),$ and the loads $M_{i,c}\left(k_{c}\right)$ are extracted only if the weight belongs to a specified force interval. This algorithm allows to extract from a complex trajectory the contribution of wheels, people, and crutches. After this operation, for each trajectory type, the algorithm calculates the first and second moments of the spatial coordinates. We experimentally found these values, depending on the trajectory types (people, wheels, pressure spots), are different for the diverse event categories and, therefore, can be compared with appropriate thresholds to eventually establish the event type.

## 3. Experimental Results

### 3.1. Characterization System

**Figure 5:**
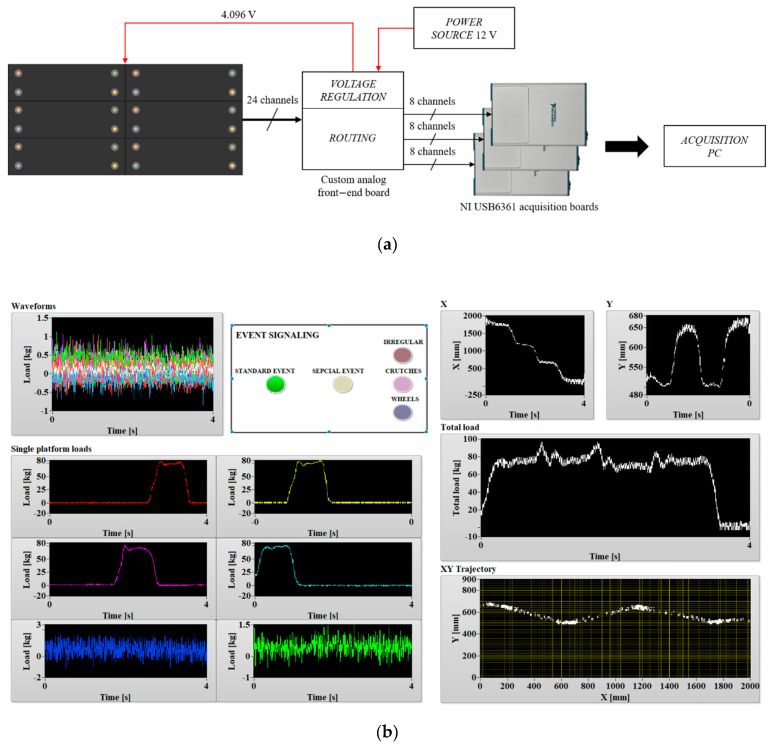
(**a**) Acquisition system block scheme. (**b**) Detail of the front panel of the LabView VI managing the acquisition and implementing the data processing.

We proved the feasibility of the system depicted in the previous section, and we characterized its performance, realizing a gravimetric apparatus made up of a gravimetric footboard prototype handled by tailored acquisition, analysis electronics and software. The gravimetric sensor prototype (produced by Saima Sicurezza S.p.a.) was an array of six 2 × 0.9 m^2^ steel plates, arranged as in Figure 1b, each laid on four load cells (range 250 kg–2 mV/V, produced by N.B.C. Elettronica Group) placed near the corners. A custom front-end circuit was designed and manufactured to power the load cells with a regulated voltage (from a 12 V power source to a 4.096 V regulated voltage reference) and to individually acquire their signals. The first realization of this circuit, that we used for the study presented in this paper, included no amplification stage in the analogic chain, but performed only the routing of each load cell signal to a different channel of one of three NI USB6361 (National Instruments) acquisition boards (16 bits A/D, 8 differential channels), which were connected to an acquisition PC. The software managing the acquisition and the real time analysis, i.e., trajectory estimation and classification, was implemented in the LabVIEW and Matlab programs, and it was capable of a 100 Hz sampling speed per channel. This frequency was sufficient for reconstructing the variable force exerted by a person walking on the footboard and his/her trajectory. Figure 5a shows a block scheme of the realized acquisition system, while Figure 5b shows the front panel of the LabVIEW VI managing the acquisition and implementing the data processing.

### 3.2. Characterization Results: Trajectory Estimation

**Figure 6:**
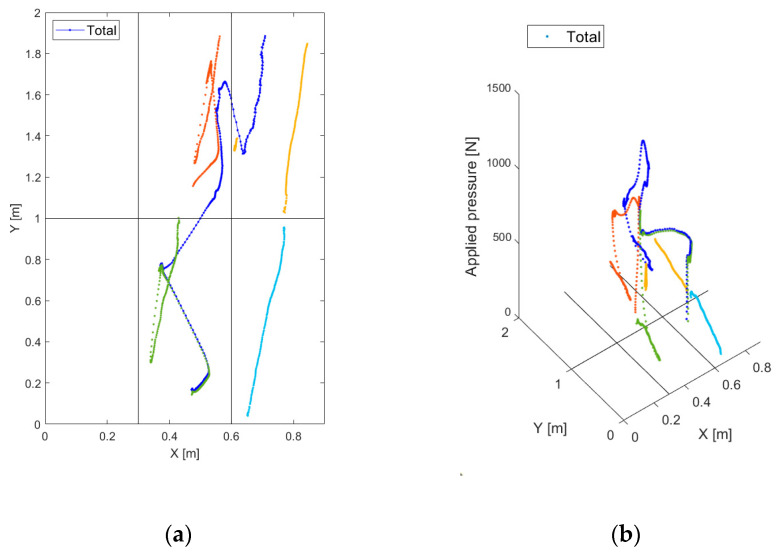
Trajectory extrapolation: case of a person pulling a luggage on wheels (**a**) 2D, (**b**) 3D.

**Figure 7:**
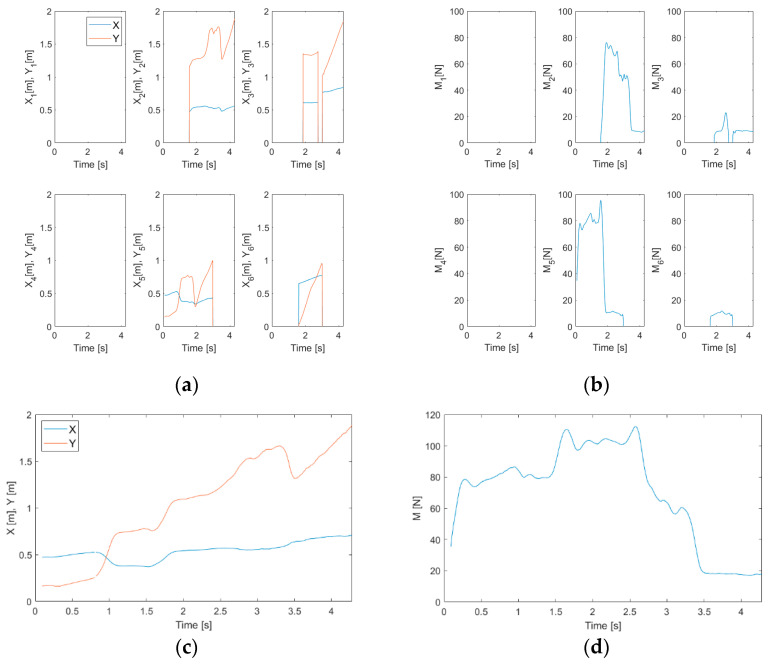
Trajectories and applied forces with respect to time: case of a person pulling a luggage on wheels. (**a**) X and Y coordinates for each footboard; (**b**) vertical force resultant magnitude for each footboard; (**c**) total X and Y coordinates; (**d**) total vertical force resultant magnitude.

Figure 6 shows an example of trajectory estimation in the case of a person pulling a luggage on wheels. Regarding both Figure 6a,b, the trajectory of the application point of the force resultant, (X(k), Y(k)), is drawn in blue, while the other colors refer to the trajectories on the individual platform ($X_{i}\left(k\right),\;Y_{i}\left(k\right)),\;i=1,\ldots ,\;6$. Figure 6b is the 3D visualization of the 2D trajectories in Figure 6a, with the measured force, M(k) and $M_{i}\left(k\right),\;i=1,\ldots ,\;6$, in newtons as the third dimension. It also is interesting to plot the load and the trajectory coordinates with respect to the time, as shown in Figure 7, for the same event of Figure 6. Figure 7a,b shows the spatial coordinates and the load magnitude, respectively, for each single platform, while Figure 7c,d show the coordinates of the resultant force application point and its intensity.

The accuracy performances of the described gravimetric system were evaluated using the method introduced in Section 2.2, applying Equations (6)–(11). The obtained trajectories and related error envelopes were similar to the results shown in Figure 3, with a maximum error on the assessment of the net force application point location of 10 cm (uncertainty 3 cm).

### 3.3. First Level Event Recognition Algorithm Results

**Figure 8:**
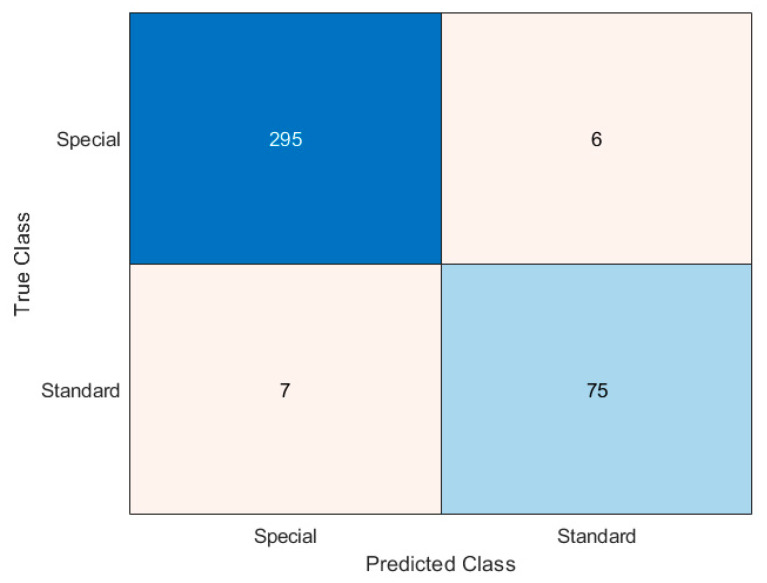
Confusion matrix summarizing first tier event recognition algorithm results.

Table 1 reports, for each tested event category, the number of test cases, the expected output for the two event recognition levels, and the expected signaling (alarm or no alarm). Figure 7c, for example, shows how the entering on the footboard by the luggage on wheels causes inversions in the Y coordinate trend (in red). These inversions are the ones called “back steps” in Section 2.4.1. Regarding the event represented in Figure 6 and Figure 7, the back steps correspond to dramatic changes in the total mean applied load, as can be determined from Figure 7d. Therefore, in this case, the first-tier event recognition algorithm is surely capable of classifying the event as “special” and passing the execution to the second-tier recognition algorithm. Figure 8 summarizes the results for the first level event recognition algorithm: the special event category, thanks to the check on both spatial coordinates, shows an excellent detectability.

### 3.4. Second Level Event Recognition Algorithm Results

**Figure 9:**
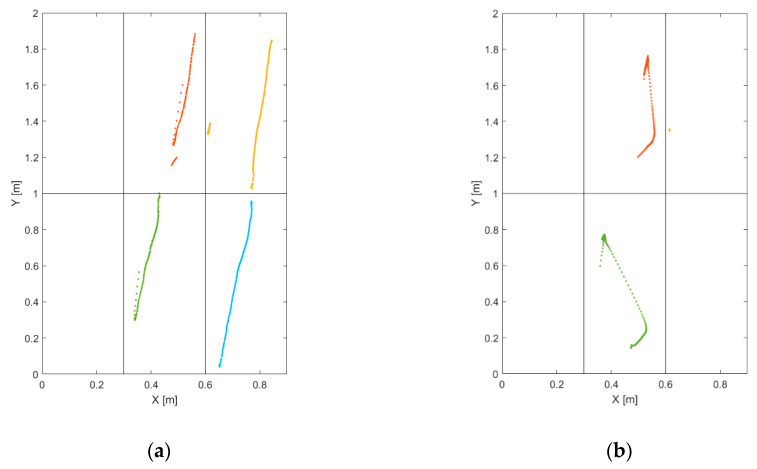
(**a**) Wheel extraction $\left(X_{i,wheel}\left(k_{wheel}\right),\;Y_{i,wheel}\left(k_{wheel}\right)\right)$ and (**b**) people extraction $\left(X_{i,people}\left(k_{people}\right),\;Y_{i,people}\left(k_{people}\right)\right)$: case of a person pulling luggage on wheels.

**Figure 10:**
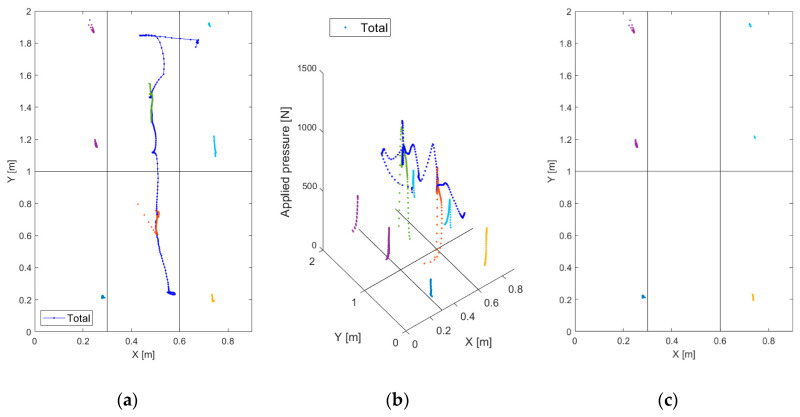
Trajectory extrapolation: case of a person on crutches (**a**) 2D, (**b**) 3D. (**c**) Load spot extraction $\left(X_{i,spot}\left(k_{spot}\right),\;Y_{i,spot}\left(k_{spot}\right)\right)$.

Figure 9 displays the results of wheel extraction (Figure 9a) and people extraction (Figure 9b) for the same event in Figure 6 and Figure 7. It is clearly visible how the extraction procedure, in this case, is capable of isolating the wheel tracks from the person tracks. Another meaningful example is given in Figure 10, which refers to the passage of a person on a pair of crutches. Concerning Figure 10, Figure 10b is the 3D representation of the 2D trajectories of Figure 10a, with the applied force in newtons as the third dimension. Furthermore, it is interesting to observe the effect of load spot extraction (Figure 10c), which is capable of evidencing the footholds of the crutches.

**Figure 11:**
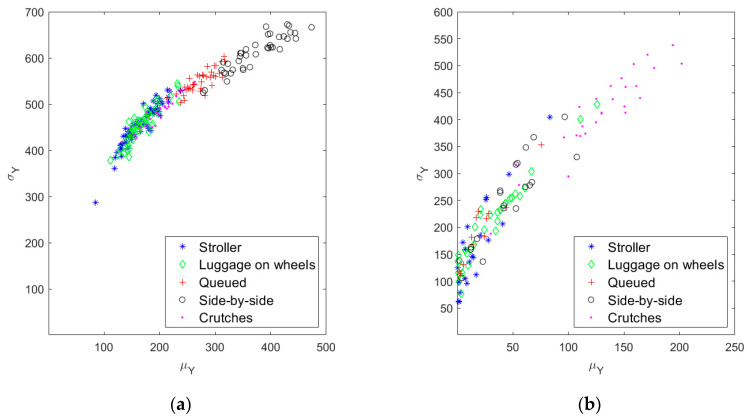
(**a**) Mean and variance of $Y_{people}$ coordinate after people extraction; (**b**) mean and variance of $Y_{spot}$ coordinate after pressure spot extraction.

As explained in Section 2.4.2, after the extraction of the different tracks, the second level event recognition algorithm calculates the mean and the variance for the spatial coordinates for each track type. Plotting these moments one versus the other reveals a remarkable differentiation of the events depending on the event type and on the performed extraction. Specifically, we found that the relevant quantities for classification are the mean and the variance of the $Y_{c}$ coordinate in the case of people extraction and in the case of pressure spot extraction, plotted respectively in Figure 11a,b. Observing these two images, it is apparent that imposing appropriate thresholds on the $Y_{people}$ coordinate moments after people extraction allows the identification of irregular events (i.e., people queued and people side-by-side), as they are rather sharply separated from the others (Figure 11a); similarly, spot extraction permits the recognition of people on crutches (Figure 11b). Consequently, the events that are categorized neither as irregular nor as crutches are labeled as “wheel events”.

**Figure 12:**
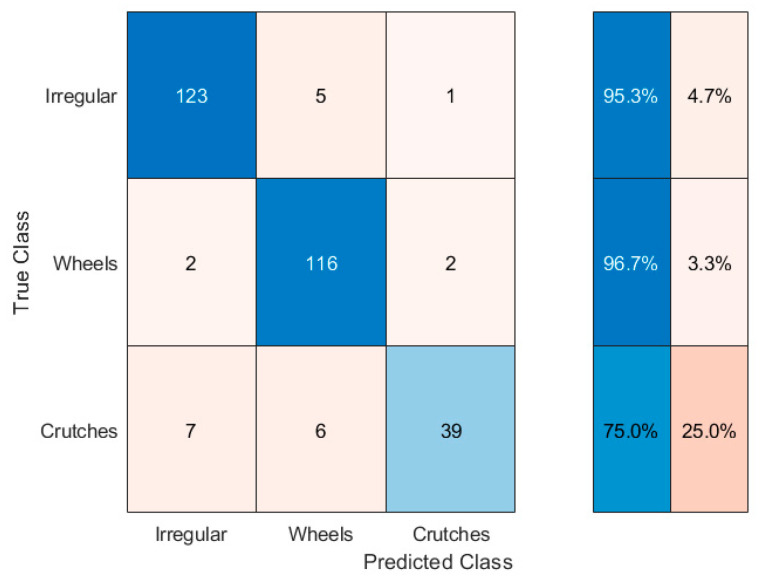
Confusion matrix summarizing second tier event recognition algorithm results.

**Figure 13:**
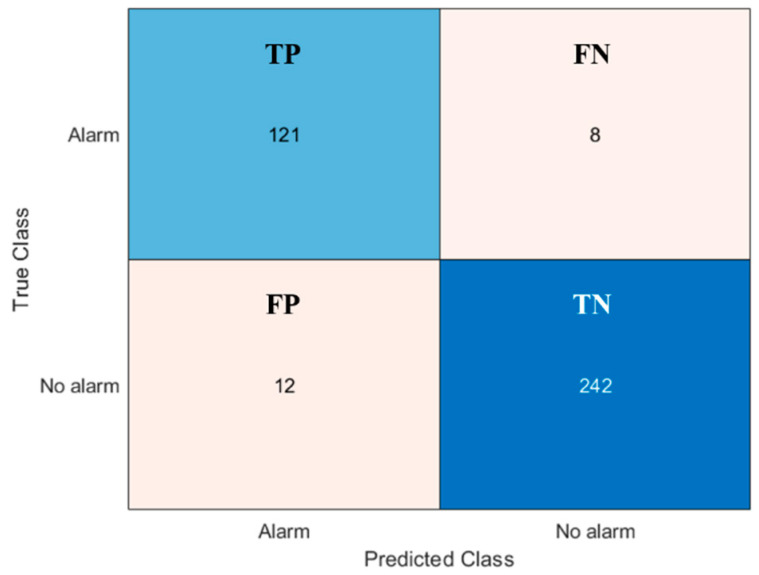
Confusion matrix summarizing the results for the entire event recognition algorithm.

The confusion matrix of Figure 12 summarizes the results for the second-tier event recognition algorithm. During this performance evaluation, we considered as input only the special events (301 in total), neglecting the errors introduced by the first step event recognition algorithm. Thus, we could assess the performance of the second-tier event recognition algorithm independently. Lastly, the confusion matrix of Figure 13 recaps the overall performance of the entire classification method, in terms of true/false positive (TP and FP) and true/false negative (TN and FN) alarms (see Table 1).

## 4. Discussion

The results presented in Section 3 are really encouraging, showing, in particular, an excellent detectability of irregularities (95.3% of all the test cases). The difference between irregular category detectability in Figure 12 and true positives in Figure 13 is due to the first level event recognition algorithm errors, that we excluded in evaluating second level event recognition algorithm performance (as stated in Section 3.3). The overall performance of the algorithm, in other words, is spoiled by the inability of the first-tier event recognition algorithm to individuate all the special events (see Figure 8).

**Figure 14:**
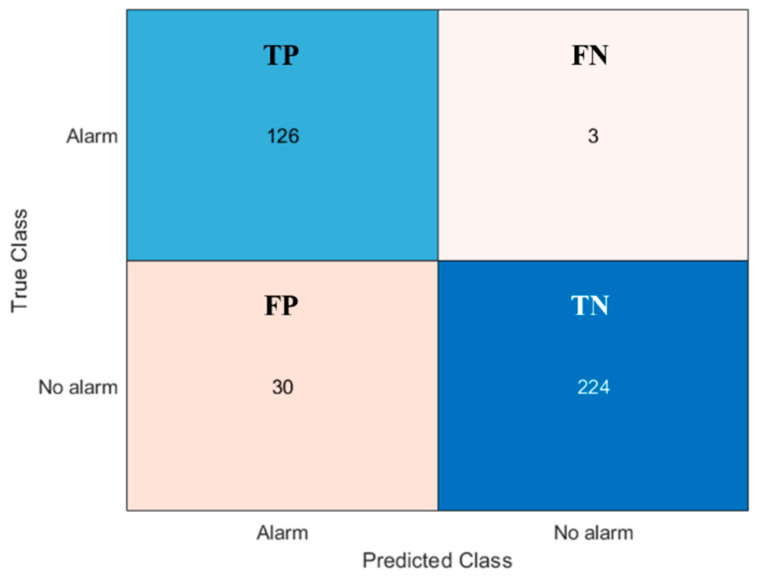
Confusion matrix summarizing the results for the entire event recognition algorithm in the case of irregular event recognition boost.

The capability of spotting the irregularities, in the application of interest, should be the most desirable; on the other hand, the occurrence of some false alarms is more acceptable than missing a security violation. Taking this perspective, the algorithm flexibility in changing the classification parameters is a key feature to adapt the system to different situations. Indeed, the parameter setting can give a boost to the recognition of a specific event category: for example, acting properly on the first level event recognition algorithm parameters, and making the thresholds more restrictive on the quantities plotted in Figure 11a for the second level event recognition algorithm, led to the results presented in Figure 14. Therein, the modified set of parameters allowed a better identification of the irregularities, bringing more frequent false alarms as a drawback. Thus, generally speaking, the choice of the algorithm thresholds and parameters is a critical task, which must be accomplished while assessing the field application of the system and deciding which error type is more tolerable.

To the best of our knowledge, the presented work is a total novel application of this kind of gravimetric system. The authors of [18] employ a similar gravimetric sensor architecture, based on a platform laid on four load cells, but applied to indoor localization of objects, people, or robots; instead, in our proposal, the aim is to recognize the signatures of different events. Found in most of the other works, more packed gravimetric sensor matrices are used, resulting in finer information but in greater complexity, with a higher cost and less robustness as well, making these devices unfeasible for the automatic entrance control of busy public places; our system, on the other hand, exploits only four sensors per platform, lowering the costs, and easing the hardware structure and the analysis software. Additionally, the load cell data processing reconstructing the trajectories, although very simple, actually builds a sort of virtual array of load cells, with the limitation of the position calculation accuracy (see the Conclusions).

## 5. The Complete Gravimetric System

**Figure 15:**
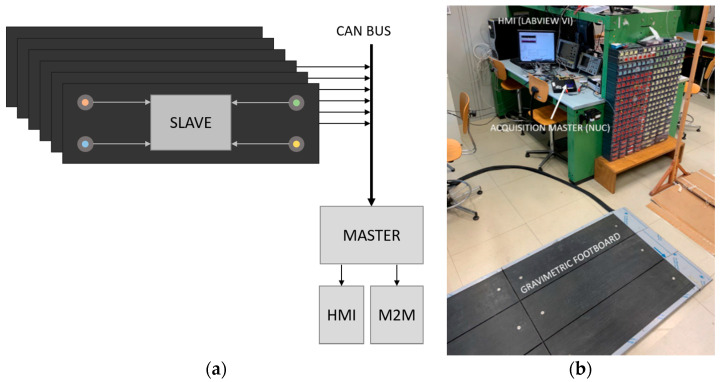
(**a**) Block diagram of the complete gravimetric system architecture. (**b**) Complete gravimetric system set-up.

Here, the overall gravimetric system architecture is described. The diagram shown in Figure 15a illustrates the architecture of the complete gravimetric system, also showing the connection between the different components of the system. Fully, each gravimetric platform (dark gray rectangles) composing the total footboard is equipped with its own analog conditioning electronics and digital signal processing capability (based on a microprocessor embedded in the slave block in Figure 15a). Each slave performs load cell signal sampling, provides on-line signal filtering, and extrapolates the force application point trajectory for the platform it serves. The as-obtained data are then sent to a central processing unit (labeled master in Figure 15a) via Controller Area Network (CAN) bus (1 Mb/s at 40 m). We provided two different communication layers at this step: a high-priority low rate data flow for pre-processed data (force and trajectory for each platform), and a low-priority high frequency data flow for transmitting raw data, if required, and for service communications (i.e., configuration parameters from the master). A PC or another microcontroller acts as the master, configuring the slaves, collecting the data from all the platforms, performing data processing, presenting the results (through a Human to Machine Interface, HMI) and making them available to other devices (Machine To Machine, M2M) in JavaScript Object Notation (JSON) format. Figure 15b shows the complete gravimetric system setup: the gravimetric footboard and the acquisition master (Intel Next Unit of Computing, NUC). Seen in the background, a part of the LabVIEW VI, acting both as an HMI (see Figure 5b) and as an M2M, is visible.

The described gravimetric apparatus demonstrates the fundamental benefit of scalability, thanks to its modular architecture. An arbitrary number of gravimetric platforms can be assembled, with almost no change in the slave block realization, and with minor modifications to the master software, granting the flexibility of the system.

Regarding the next sections, the slave and master hardware and software architectures are described in detail. The adopted policy for the communication between the master and the slaves via CAN bus is reported in a separate section.

### 5.1. Slave Architecture

Each gravimetric platform is equipped with a circuitry which is called slave in this discussion. The slave hardware is composed of two main blocks: an analog front-end and a Cortex-M4 based STM32F373 (STMicroelectronics) microcontroller mounted on a readapted Nucleo-64 board. The analog conditioning circuit is made up of four identical channels (one per each load cell) providing signal filtering and amplification. The amplification is conveniently set to match the input range of the 16-bit sigma-delta converter present on the microcontroller. Occurring at the system start-up, the microcontroller reads some configuration parameters from a dedicated area of its on-board non-volatile memory, which also is accessible to the master via CAN bus (see Section 5.3 for details on the configuration parameters). The microcontroller samples the ADC output at a frequency called the “sampling frequency”, averages the acquired data reducing the sampling frequency to a lower one, called the “update frequency” (the data obtained at this point are called “raw data”), calculates the resultant force magnitude ($M_{i}$) and application point position ($X_{i}$, $Y_{i}$), and sends this processed information through the CAN bus. This main routine is executed by each slave microcontroller continuously. Each slave microcontroller, in other words, independently presents the samples of the three signals ($M_{i}$, $X_{i}$, $Y_{i}$) on the CAN bus at the update frequency, with no need for a request from the master. Additionally, each slave listens for configuration parameter changes from the master.

### 5.2. Master Architecture

**Figure 16:**
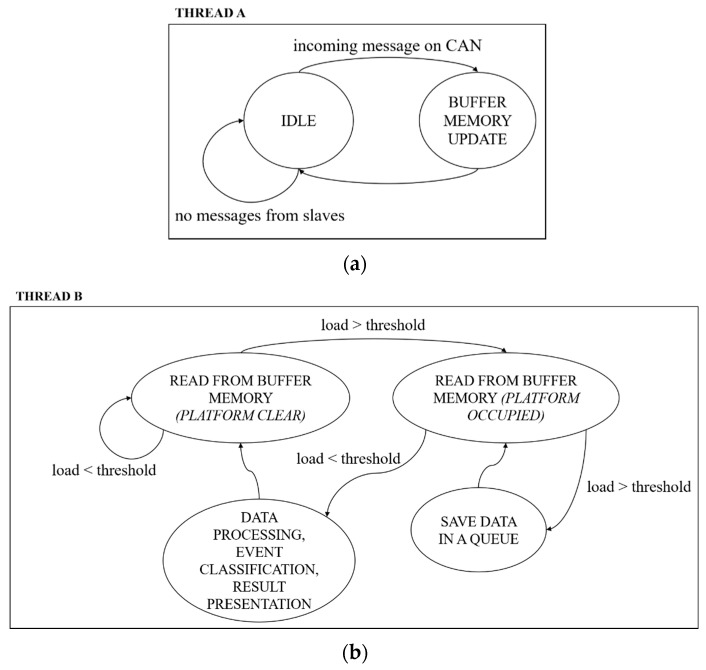
Master software architecture: (**a**) thread A, (**b**) thread B.

**Figure 17:**
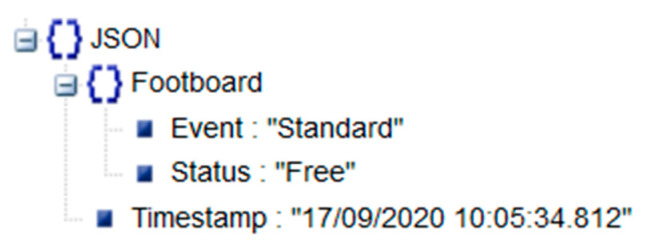
JSON output (seen in JSON viewer) in case of a standard event.

The role of the master in the proposed system is played by an Intel NUC. The master configures the slaves, collects the information from all the platforms, performs data processing and produces the final output (i.e., event categorization, Human to Machine Interface (HMI) and Machine To Machine (M2M) interfaces). The master software consists of two separate threads working in parallel, as illustrated in Figure 16. Thread A (Figure 16a) is the CAN bus reading thread: it waits in an idle state until a new message (i.e., a platform state update consisting of the three new samples of $M_{i}$, $X_{i}$, $Y_{i}$) from one of the slaves is available on the CAN bus; when a slave sends a platform status update, it is stored in a buffer memory, which represents the current overall footboard status; then, the thread A goes back into the idle state. Thread B (Figure 16b) is the data processing thread: it reads the footboard status from the buffer memory at the update frequency (PLATFORM CLEAR state) and compares the total load charging the footboard with a load threshold (see Section 2.4); if the total load is over the threshold, the master continues reading from the buffer memory (and switches to PLATFORM OCCUPIED state), but stores the data in a queue rather than discarding them; when the footboard is recognized to be clear again (total load under the threshold), the data queue is passed to the data processing and event classification algorithm (see Section 2.4) to produce the final output, which is presented to the user and exposed via TCP/IP in JSON format (Figure 17 reports an example of JSON output for a standard event).

### 5.3. CAN Communication

A CAN frame has 11 bits for the message identifier and up to 8 bytes for the data payload. During our CAN bus implementation, we reserved 8 message identifiers per each platform (slave). This means that a maximum of 256 platforms can be connected simultaneously in our system. The message identifier, therefore, is composed of a base address, that is the platform identifier, settable by means of dip switches mounted on the slave boards, and of an offset from 0 to 7 determining the message type. The meanings of the 8 possible message types are listed in Table 2. Occurring at the system start up, the master sends the configuration to all the slave platforms, namely the sampling frequency, the update frequency (see Section 5.1), the number of connected load cells (we added this option for flexibility, to be able to also connect gravimetric platforms with a different number of sensors), and a mask for excluding cells from the acquisition in case of faults. This last option is particularly useful in security applications, where faults must be addressed without interrupting the service. Once the configuration is complete, the platforms begin to send their pre-processed data ($M_{i}$, $X_{i}$, $Y_{i}$), as described in Section 5.1, using the offset message identifier 0 (high priority). The debug mode, activatable with offset 5, instructs the platforms to send the raw data (see Section 5.1) using the high speed CAN data layer (see Section 5 introduction). The raw data are sent using offset 7. Thus, the raw data are given the lowest priority. This way, even if the CAN bus is congested with raw data communications, it is still possible to force off the debug mode thanks to the higher priority of the offset 5.

## 6. Conclusions

Here, we presented a low cost, robust, and flexible gravimetric system for access monitoring applications, consisting of an array of simple gravimetric footboards based on load cells. The operations of the system were described and analyzed from a theoretical point of view and validated by means of a data acquisition set-up.

Considering the presented results, it can be seen that, despite its simplicity and the small number of sensors used, the system was able to reconstruct the trajectory of the application point of the vertical component of the applied force, resulting in high accuracy.

Regarding the presented system implementation devoted to public place access control, the typical accuracy is in the order of centimeters. Generally, the spatial accuracy is related to the ratio between the magnitude of the net vertical force itself and the load cell uncertainty; therefore, in most applications, a satisfactory accuracy can be obtained through a fit selection of the sensors.

Furthermore, the trajectory on the complete footboard is split into the elementary trajectories on the individual platforms. This allows a refinement in the knowledge about the spatial distribution of the forces applied on the overall footboard, and to gain more information about the transit occurring on the footboard. Here, the proposed gravimetric system has been applied to access control in public places, but it also can be applied in other fields by adapting the size and number of platforms. Gait analysis, for example, represents a possible area of employment: in fact, although our gravimetric system is not capable of drawing a detailed force map of the feet, it can extract the trajectory and the intensity of the force exerted on the lower limbs.

An ad hoc trajectory analysis algorithm was presented, aimed at an on-line identification of irregular events, i.e., multiple and simultaneous transits. Considering this, the experimental results showed good performance in detecting multiple people stepping simultaneously on the gravimetric footboard, both side-by-side and queued. This type of event is generally relevant to access control activities and is crucial if the goal is to perform these activities automatically. Other types of events, such as people with wheeled luggage or on crutches, have been investigated with satisfactory results. Correct recognition of these patterns also could be relevant to minimize false positives in security checks performed with heterogeneous technologies such as metal detectors, video analysis, etc., allowing a data fusion approach for non-invasive checks in public spaces.

The implemented event recognition algorithm can be further improved in future development of this work. Particularly, a Machine Learning (ML) approach can be introduced to refine the accuracy of the event classification; moreover, thanks to ML, it might be possible to add to the event category set other likely occurrences, currently hard if not impossible to spot, such as the passage of a person on a wheelchair.

Finally, it must be underlined that, to the authors’ knowledge, all the other approaches for safety applications are based on dense matrices of sensors embedded in a floor. The proposed approach has the peculiarity of combining a ‘real’ but very simple sensor array (the six platforms) with a ‘virtual’ array (the single platform) created by exploiting the torque equilibrium. The ‘virtual’ array is able to reach a very fine resolution (as if having a very large number of sensing elements), which is limited only by the sensor uncertainty. The virtual array performance, nonetheless, is limited because the obtainable information is related to the ‘resultant’ force on the individual platform, therefore the virtual array works very well in the case of a single force application point but has a limited performance in the case of multiple application points, and it can be used only in conjunction with advanced signal processing techniques or artificial intelligence to unravel the condensed information.

## Figures and Tables

**Table 1:** Test cases and expected behavior of the classification algorithm.

Event Category	Description	Expected First Tier Classifier Output	Expected Second Tier Classifier Output	Expected Signaling	Test Cases
Standard event	Single person without any particular object	STANDARD	-	NO ALARM	82
Wheel event	Single person with luggage on wheels or with a stroller	SPECIAL	WHEELS	NO ALARM	120
Crutches event	Single person on crutches	SPECIAL	CRUTCHES	NO ALARM	52
Irregular event	Two people side-by-side or two or more people queued	SPECIAL	IRREGULAR	ALARM	129

**Table 2:** CAN Message types.

Offset	Meaning	From Master to Slaves	From Slaves to Master
0	Pre-processed data (M, X, Y)		*X*
1	Sampling frequency	*X*	
2	Update frequency	*X*	
3	Number of connected load cells	*X*	
4	Mask of functioning cells	*X*	
5	Debug mode	*X*	
6	Not used (left for future use)		
7	Raw data (only in debug mode)		*X*
